# Dental caries remains as the main oral condition with the greatest impact on children’s quality of life

**DOI:** 10.1371/journal.pone.0185365

**Published:** 2017-10-05

**Authors:** Milene T. Martins, Fernanda Sardenberg, Cristiane B. Bendo, Mauro Henrique Abreu, Míriam P. Vale, Saul M. Paiva, Isabela A. Pordeus

**Affiliations:** 1 Department of Pediatric Dentistry and Orthodontics, School of Dentistry, Federal University of Minas Gerais, Belo Horizonte, Brazil; 2 Department of Pediatric Dentistry, School of Dentistry, Estadual University of Montes Claros, Montes Claros, Brazil; 3 Department of Social and Preventive Dentistry, Faculty of Dentistry, Federal University of Minas Gerais, Belo Horizonte, Brazil; INDIA

## Abstract

**Purpose:**

The objective of this study was to assess the negative impact of dental caries on the OHRQoL of 8- to 10-year-old Brazilian children.

**Methods:**

This population-based case-control study involved 546 children (8–10 years old), 182 cases with a high negative impact on OHRQoL and 364 controls with a low negative impact on OHRQoL. Children’s OHRQOL was measured using the Child Perceptions Questionnaire (CPQ_8-10_). Cases and controls (1x2 ratio) were individually matched by school and gender. Dental caries experience, malocclusion, and traumatic dental injuries were used as independent variables. Dental examinations were carried out at school during daytime hours by two calibrated examiners (Kappa = 0.93-interexaminer and 0.95- intraexaminer). The data were analyzed by descriptive statistics, conditional bivariate and multiple logistic regression, with the significance level set at 5%.

**Results:**

There was no significant difference in traumatic dental injuries and malocclusion between the case and control groups (p>0.05). Children with DMFT/dmft ≥3 had a 2.06-fold (95%CI = 1.28–3.31, p = 0.003) greater chance of experiencing a high negative impact on OHRQoL than those with DMFT/dmft = 0

**Conclusion:**

Children with high dental caries experience are more likely to present a high negative impact on OHRQoL than those with no dental caries experience.

## Introduction

Dental caries is a public health problem in Belo Horizonte, state capital of Minas Gerais (southeastern Brazil) [1, 2], as well as in other Brazilian cities [3,4], and in many countries [5]. In 2010, untreated caries in permanent teeth was the most prevalent health condition worldwide, affecting 2.4 billion people, and untreated caries in deciduous teeth was the 10th most prevalent health condition, affecting 621 million children worldwide [6]. In Brazilian children, data from the 2010 Oral Health Project reveal that 56.5% of 12-year-olds have at least one permanent tooth with dental caries experience. This represents approximately 1.7 million children [7]. Brazilian children also have a high prevalence of malocclusion (38.8%) and traumatic dental injury (20.5%) up to the age of 12 years [7].

In recent years, these conditions have been associated with a negative impact on children’s quality of life [8, 9, 10, 11]. Cross-sectional studies [12, 13, 14] demonstrated that dental caries have been associated with a negative impact on the quality of life of children from different age groups [15, 16, 17] So far, only one study, including 3- to 5 year-old children [18] had used a case-control design to evaluate the association between dental caries and negative impact on oral health-related quality of life (OHRQoL). A case-control study provides adequate statistical power. This power depends not only on the number of cases and controls, but also on the distribution of the exposure of the population at risk and on the relative risk of disease the study aims to detect [19]. Instead of measuring relative risk of disease based on exposure, a retrospective case-control study allows us measure the odds of exposure based on disease.

Given the paucity of case-control studies on the impact of oral conditions on OHRQoL, the objective of this study was to assess the negative impact of dental caries on the OHRQoL of 8- to 10-year-old Brazilian children, controlled by the other two main oral conditions in children (malocclusion and traumatic dental injury), using a design that offers greater strength of evidence.

## Materials and methods

### Study design

A population-based case-control survey was carried out with a representative sample (546 male and female children aged 8 to 10 years, attending public and private elementary schools) in the city of Belo Horizonte, Brazil. Belo Horizonte is divided into nine regional areas of local administration. This case-control study was nested in a cross-sectional study [20].

A multistage sampling technique was adopted to select children. The first stage was comprised of randomly selected public and private elementary schools in each administrative district of Belo Horizonte. In the second stage, classes were randomly chosen from the selected schools.

### Sample size calculation

The sample size was calculated to give a power of 80.0% and a standard error of 5.0%. Two controls were individually matched for each case. The odds ratio (OR) used was set at 2.0 and the probability of dental caries experience among cases was set at 63.7% [10]. The probability of traumatic dental injuries among cases was set at 16.0% [21] and the probability of malocclusion among cases was set at 57.2% [10]. The sample size was calculated for each oral condition. The probability of traumatic dental injuries among cases was used due to their lower prevalence, indicating the need for a larger sample. The minimum sample size to satisfy the requirements was 182 cases and 364 controls.

### Dependent variable

Oral health-related quality of life (OHRQoL) was the dependent variable. The Brazilian version of the Child Perceptions Questionnaire for ages 8 to 10 years (CPQ_8-10_) was used [22] to assess the impact of oral conditions on OHRQoL. The CPQ_8-10_ is an OHRQoL instrument designed exclusively for this age group. This instrument has been proven to be valid and reliable for use in Brazilian children [22]. This instrument is made up of 25 items distributed into four subscales: oral symptoms (5 items), functional limitations (5 items), emotional well-being (5 items), and social well-being (10 items). The items address the frequency of events in the four previous weeks. A five-point rating scale is used, with the following options: never = 0; once/twice = 1; sometimes = 2; often = 3, and every day/almost every day = 4. CPQ_8-10_ scores are calculated by summing all the item scores, with the total score ranging from 0 (no impact of oral condition on OHRQoL) to 100 (maximum negative impact of oral condition on OHRQoL).

In order to define cases and controls, the CPQ_8-10_ scores were analyzed using the two-step cluster method. The log-likelihood distance measure was used. Two-step cluster analysis considered the 25 items of the Brazilian version of the CPQ_8-10_ separately and compared the mean of one item within each cluster to the overall mean of the same item in the total sample [23]. The case group included those children who experienced a higher negative impact on OHRQoL whereas controls showed a lower negative impact on OHRQoL. Cases and controls were individually matched by school and gender.

### Independent variables

Dental caries experience was diagnosed by the Decayed, Missing and Filled Teeth Index (DMFT∕dmft) [24]. Dental caries experience was divided into three groups: children with DMFT∕dmft = 0, children with DMFT∕dmft 1 or 2, and children with DMFT∕dmft ≥3.

The Dental Aesthetic Index (DAI) criteria [25] were used to measure malocclusion, which was dichotomized as either absent/mild (DAI ≤ 25) or present (DAI > 25).

Traumatic dental injury in permanent incisors was recorded based on the criteria proposed by Andreasen (2007) [26]. Children were classified as with or without TDI.

### Data collection

Dental examinations were carried out at schools during daytime hours, in a private room. The examiners were seated in front of the children, who remained seating. The examiners used appropriate equipment to protect against individual cross-infection, with all instruments sterilized. The CPQ_8-10_ was applied by interviews before the clinical exams.

During the calibration process, 70 children (5.8% of the sample and not part of the study population) from a convenience sample were examined by two dentists. The examiners assessed the three conditions (dental caries experience, malocclusion, and TDI) and obtained inter-examiners agreement (kappa = 0.78–1.00). They re-examined the children after two weeks to assess intra-examiners agreement (kappa = 0.93–1.00).

### Data analysis

The Statistical Package for Social Sciences, version 19.0 (SPSS Inc., Chicago, IL, USA), was used for the statistical analysis. Data analysis involved descriptive statistics. Bivariate logistic regression analysis was conducted to measure the association between independent variables and the negative impact on OHRQoL. Multiple conditional logistic regression was used for the matched case–control study. All the three clinical conditions were included in the logistic model based on their clinical epidemiological importance. The significance level was set at 5%.

### Ethical approval

The research was ethically conducted in accordance with the Declaration of Helsinki. The study was approved by the Human Research Ethics Committee of the Federal University of Minas Gerais (protocol 04.65.0.203.000–09). Parents/guardians and children read and signed an informed consent form prior to their participation in the study.

## Results

This population-based case-control study involved 546 children, 182 cases with a high negative impact on OHRQoL and 364 controls with a low negative impact on OHRQoL. The mean age was 9,08 (29.7%- 8 years old; 32.6%- 9 years old and 37.7%- 10 years old). The controls were individually matched with cases for gender and school using a 2:1 ratio. Table 1 demonstrates the frequency distribution of independent variables for matched cases and controls. There was no significant difference in traumatic dental injuries and malocclusion between the case and control groups (p>0.05). There was a statistically significant difference in dental caries experience between the case and control groups (p<0.05).

**Table 1:** Frequency distribution of independent variables for matched case and control groups (n = 546).

Variables	Case group (n = 182) n (%)	Control group (n = 364) n (%)	Unadjusted OR (95% CI)	P-value ^a^
**Traumatic dental injuries**				
Absent	156 (85.7)^b^	314 (86.3) ^b^	1.00	
Present	26 (14.3) ^b^	48 (13.2) ^b^	1.09(0.65–1.82)	0.742
Missing		2 (0.5)^C^		
**Malocclusion**				
Absent	119 (65.4) ^b^	257 (70.6)^b^	1.00	
Present	62 (34.1) ^b^	106 (29.1) ^b^	1.26 (0.86–1.85)	0.230
Missing	1 (0.5) ^C^	1 (0.3) ^C^		
**Dental caries**				
CPOD/ceod = 0	88 (48.3) ^b^	230 (63.2) ^b^	1.00	
CPOD/ceod = 1 ou 2	50 (27.5) ^b^	81 (22.3) ^b^	1.61 (1.05–2.47)	0.029
CPOD/ceod = 3 a 10	44 (24.2) ^b^	53 (14.5) ^b^	2.17 (1.35–3.46)	0.001

^a^ Bivariate conditional logistic regression.

^b^ Values in parentheses refer to the percentages in the columns.

^c^ Missing values during the data collection

Table 2 displays the multiple conditional logistic regression and the influence of dental caries experience on children’s OHRQoL. Although traumatic dental injuries and malocclusion did not achieve statistical significance, these variables were maintained in the model to control for potential confounding factors. The results indicate that children with DMFT/dmft 1 or 2 had a 1.61-fold (95%CI = 1.05–2.49, p = 0.029) and children with DMFT/dmft ≥ 3 had a 2.06-fold (95%CI = 1.28–3.31, p = 0.003) greater chance of experiencing a high negative impact on OHRQoL than those with DMFT/dmft = 0.

**Table 2:** Multiple conditional logistic regression model^a^ explaining the influence of dental caries on children’s OHRQoL in a matched case-control analysis (n = 546).

Variables	Adjusted OR (95% CI)	P-value ^a^
**Traumatic dental injuries**		
Absent	1.00	
Present	1.11 (0.66–1.87)	0.691
**Malocclusion**		
Absent	1.00	
Present	1.21 (0.82–1.78)	0.327
**Dental caries**		
CPOD/ceod = 0	1.00	
CPOD/ceod = 1 ou 2	1.61 (1.05–2.49)	0.029
CPOD/ceod = 3 a 10	2.06 (1.28–3.31)	0.003

^a^ Power of the study explanation was 0,894 (Hosmer and Lemeshow test)

## Discussion

Dental caries can influence children’s quality of life in activities such as eating, sleeping and talking, as well as their general health [15, 27]. The results of this study demonstrate that children with dental experience (DMFT/dmft 1 to 10) are more likely to suffer a high negative impact on their OHRQoL than those without dental caries experience (DMFT/dmft = 0). This finding is important because it proves that public health policies should be directed towards the most vulnerable groups. In Brazil, 20% of the school population concentrates about 60% of the disease burden [28].

Despite the overall reduction in the DMFT index and an increase in the prevalence of caries frees among Brazilian adolescents, there were increases in both income and education-related inequalities among caries-active individuals [29]. This indicates inequality, i.e., the different prevalence of dental caries among individuals can be explained not only by inevitable biological changes, but also by differences in the social environment in which these individuals live, which is expressed through the health-disease process [28]. More severe oral changes are found in a small percentage of the population, which requires greater attention. The damage and unequal distribution of dental caries can be minimized by comprehensive dental care, including prevention, oral health promotion, and treatment [30].

The Brazilian version of CPQ_8-10_ was used in this study to assess the impact of oral conditions on quality of life. This OHRQoL instrument was designed exclusively for this age group. This instrument has been proven valid and reliable for use in Brazilian children [22]. In order to define cases and controls, the CPQ_8-10_ scores were analyzed using the two-step cluster method. Cases and controls were individually matched by school because a previous study showed that dental caries experience and severity of dental caries in primary and permanent teeth are influenced by the type of school [20]. In Brazil, economically underprivileged children are enrolled public schools, had a higher prevalence of dental caries and the greatest impact thereof on their quality of life [16]. Cases and controls were individually matched by gender to control the perception of negative impact on quality of life in this age group between sexes, since most studies demonstrated such association [12, 16, 31]. Even though the literature shows that there is no significant difference between boys and girls as to caries increment in the mixed dentition [32].

There was no significant difference in traumatic dental injuries between the case and control groups, probably because most of the children had mild dental trauma (e.g., enamel fractures). At this age, only severe trauma is associated with a negative impact on quality of life [33].

There was no significant difference in malocclusion between the case and control groups, contradicting a study with same-age children, which found that schoolchildren with malocclusion were 1.30-fold more likely to experience a negative impact on OHRQoL than those without malocclusion [10]. This can be due to the cutoff point of the DAI. Malocclusion was dichotomized as either absent/mild (DAI ≤ 25) or present (DAI > 25). Thus, moderate malocclusions (DAI 26 to 30) could not have an impact on OHRQoL.

The present study has limitations. The sample selection used multistage sampling method rather than random sampling. This sampling method provides a cluster with children similar to each other and different from children in other clusters. For correct the similarity within the same cluster (school), a design factor of 1.2 was applied in the sample size calculation for the cross-sectional study in which this present case-control study was nested [20].

In general, there is a paucity of studies on dental caries with children in the mixed dentition phase [12, 20, 31]. In Brazil, a study using the same instrument was developed with 112 poor 8- to 10-year-old children. This previous study demonstrated that children with untreated dental caries have a greater prevalence ratio of having a negative perception of their oral health status than those without dental caries [12]. The present study has a clear advantage over this previous study because of its case-control design. It provides stronger evidence than cross-sectional studies. There is a case-control study on dental caries and quality of life; however, it evaluated 415 children aged 3–5 years enrolled in public and private preschools. This previous study showed that caries severity impacted the OHRQoL of preschool children [18]. The present study demonstrated that higher caries experience was associated with a greater negative impact on quality of life. In this way, children in the mixed dentition stage with high caries experience must be one of the priorities in the planning and implementation of public health policies for the prevention, control, and treatment of this disease.

## Conclusions

1. Children with dental experience (DMFT/dmft 1 to 10) are more likely to suffer a high negative impact on their OHRQoL than those without dental caries experience (DMFT/dmft = 0), controlled by malocclusion and traumatic dental injuries.

## Supporting information

S1 Database (182 casos e 364 controles.sav) was made available for plos one. (SAV)
